# Supplementary material for: Heat Shock 70 kDa Protein Cognate 3 of Brown Planthopper Is Required for Survival and Suppresses Immune Response in Plants
Source: Insects. 2022 Mar 17;13(3):299. doi: 10.3390/insects13030299 (PMC8949815; doi:10.3390/insects13030299)
Supplement: Supplementary file 1 [file insects-13-00299-s001.zip › Supplementary Files/Table S1.pdf]

Table S1 List of qRT-PCR primers used in this study.

Primer Name	Sequence
*Nl*Hsp70-2F	GGGGACAAGTTTGTACAAAAAAGCAGGCTTCATGAAACTAGAATTAATTGGC
*Nl*Hsp70-2R	GGGGACCACTTTGTACAAGAAAGCTGGGTCAAGCTCGTCCTTGAGATCGTC
*Nl*Hsp70-2F-D	GGGGACAAGTTTGTACAAAAAAGCAGGCTTCATGGCGGATGAAGAGAAAGGA
q*Nl*Hsp70-2R	AGCACATTCTTCTCGCCCTC
β-actin-F	GACAGGATGCAGAAGGAAATCA
β-actin-R	GACTCGTCGTACTCCTGCTTTG
*Nl*Hsp70-2-RNAi-F	ATGAAACTAGAATTAATTGGC
*Nl*Hsp70-2-RNAi-R	AAGCTCGTCCTTGAGATCGTC
q*Nb*PR1-F	TGAGATGTGGGTCGATGAGA
q*Nb*PR1-R	CGAGTTACGCCAAACCACTT
q*Nb*PR2-F	CAATGCATTAGCAGCAGCAG
q*Nb*PR2-R	ATCTTTGGGCGGGTAGGTAT
q*Nb*PR3-F	TGGGGTTATTGCTGGCTTAG
q*Nb*PR3-R	GGGTCATCCAAAACCAGAGA
q*Nb*PR4-F	GGCCAAGATTCCTGTGGTAGAT
q*Nb*PR4-R	CACTGTTGTTTGAGTTCCTGTTCCT
q*Nb*Actin-F	CGGAATCCACGAGACTACATAC
q*Nb*Actin-R	GGGAAGCCAAGATAGAGC
T7	TAATACGACTCACTATAGGGAGA
