# Supplementary material for: Heat Shock 70 kDa Protein Cognate 3 of Brown Planthopper Is Required for Survival and Suppresses Immune Response in Plants
Source: Insects. 2022 Mar 17;13(3):299. doi: 10.3390/insects13030299 (PMC8949815; doi:10.3390/insects13030299)
Supplement: Supplementary file 1 [file insects-13-00299-s001.zip › Supplementary Files/Table S2.pdf]

Table S2 The Hsp70 family members in BPH and OsHSP70 and OsHSP71.1 of rice.

Protein	GenBank accession number
Nilaparvata lugens heat shock 70 kDa protein cognate 3 (NlHSP70-3)	XM_022337451.1
Oryza sativa heat shock protein 70 (OsHSP70)	XP_015616495.1
O.sativa heat shock protein cognate 1 (OsHSP71.1)	BAG93163
N. lugens heat shock 70 kDa protein cognate 2 (NlHSC70-2)	KU932402.1
N. lugens heat shock 70 kDa protein cognate 5 (NlHSC70-5)	XM_022345825.1
N. lugens heat shock 70 kDa protein cognate (NlHSC70)	ADE34170.1
N. lugens heat shock protein 70 (HSP70)	AFJ20626.1
