# Supplementary figures and images for: Heat Shock 70 kDa Protein Cognate 3 of Brown Planthopper Is Required for Survival and Suppresses Immune Response in Plants
Source: Insects. 2022 Mar 17;13(3):299. doi: 10.3390/insects13030299 (PMC8949815; doi:10.3390/insects13030299)

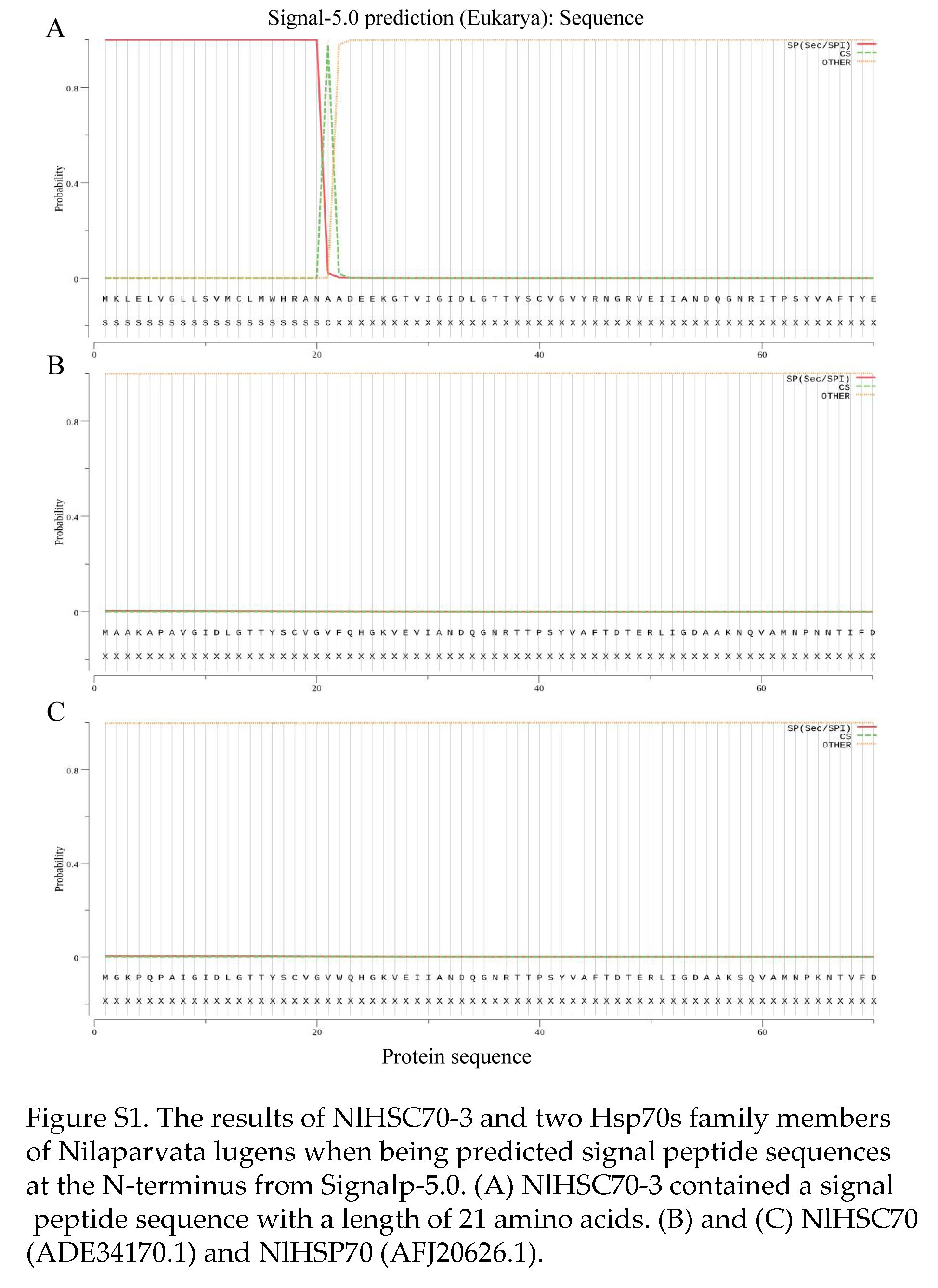

Supplement: Supplementary file 1 [file insects-13-00299-s001.zip › Supplementary Files/Figure S1.tif]

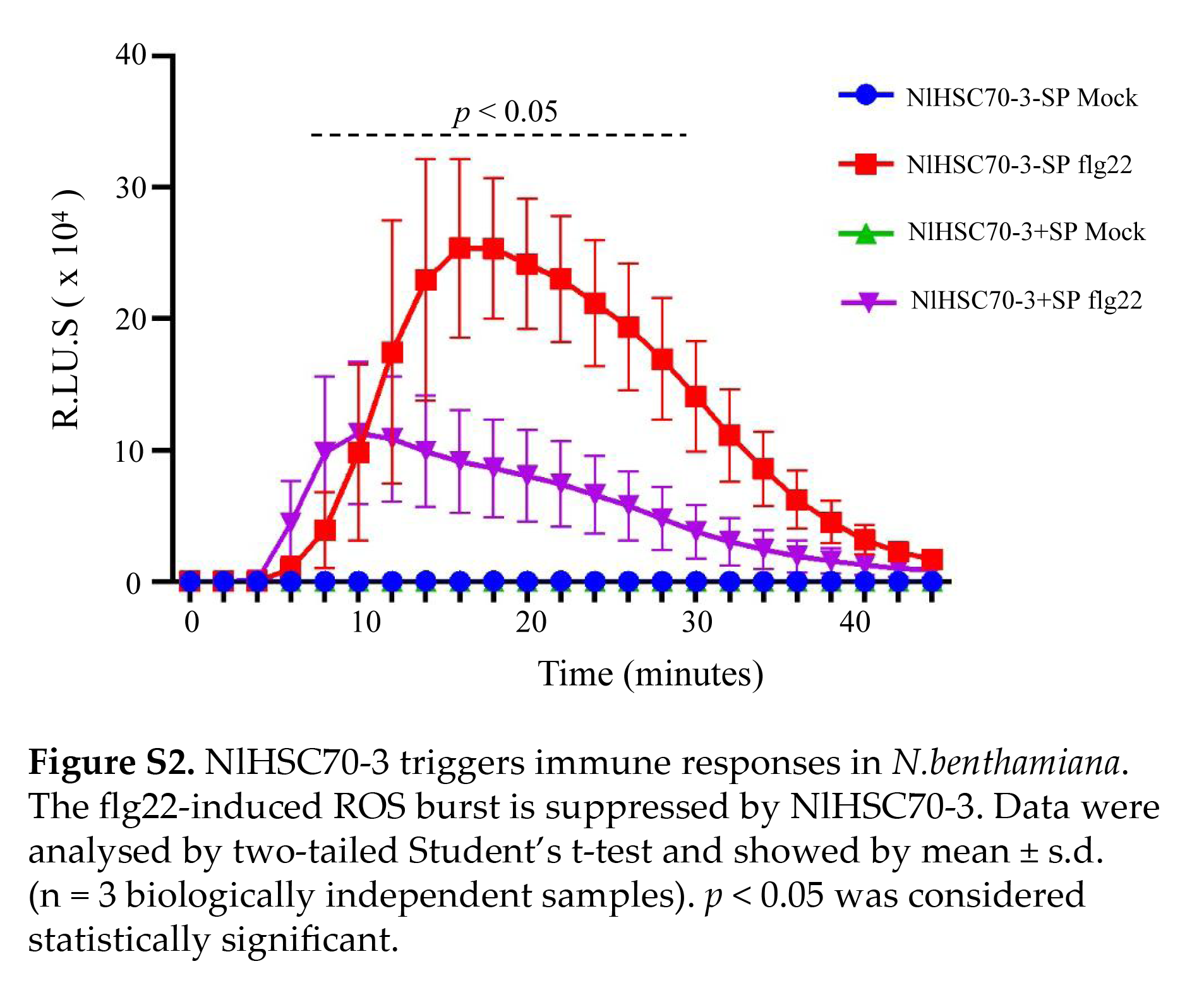

Supplement: Supplementary file 1 [file insects-13-00299-s001.zip › Supplementary Files/Figure S2.tif]
